# Clash of the pandemics – At least 150’000 adults in Switzerland suffer from obesity grades 2 or 3 and are thus at elevated risk for severe COVID-19

**DOI:** 10.12688/f1000research.27819.1

**Published:** 2020-12-07

**Authors:** Kaspar Staub, Katarina L. Matthes, Frank Rühli, Nicole Bender

**Affiliations:** 1 Institute of Evolutionary Medicine, University of Zurich, Zurich, Switzerland; 2 Zurich Center for Integrative Human Physiology, University of Zurich, Zurich, Switzerland; 3 Swiss School of Public Health SSPH+, Zurich, Switzerland

**Keywords:** NCD, Adiposity, Public Health, Risk groups, Sars-Cov-2

## Abstract

**Background**: Grade 2 and 3 obesity, alongside with other relevant risk factors, are substantially and independently associated with adverse outcomes of coronavirus disease 2019 (COVID-19). However, for Switzerland, due to the lack of synthesis studies, it is currently unknown how many people are affected by obesity at all. This knowledge may help to better estimate the relevance and size of this group at elevated risk, which could be incorporated into strategies to protect risk groups during the still unfolding COVID-19 pandemic. This study aimed to provide a first overall estimation of how many people in Switzerland are currently affected by grade 2 or 3 obesity.

**Methods**: Five representative national population-based studies were accessed which were conducted between 2012 and 2017 and which include data on height and weight of adult men and women in Switzerland.

**Results**: In Switzerland in 2012-2017, among the 11.20% adults who were obese (body mass index (BMI) ≥30.0kg/m2), 1.76% (95% CI 1.50-2.02) suffered from grade 2 obesity (BMI 35.0-39.9 kg/m2), and 0.58% (95% CI 0.50-0.66) from grade severe 3 obesity (BMI ≥40.0 kg/m2). Converted into estimated absolute population numbers, this corresponds to a total of approximately n=154,515 people who suffer from grade 2 or 3 obesity (n=116,216 and n=38,298, respectively).

**Conclusions**: This risk group includes many younger people in Switzerland. The number of people with obesity-related risk becomes 3.8 to 13.6 times higher if grade 1 obesity and overweight people are also included in this risk group, for which there are arguments arising in the latest literature. In general, this large group at risk for severe COVID-19 should be given more attention and support. If it is confirmed that obesity plays a major role in severe COVID-19 courses, then every kilo of body weight that is not gained or that is lost in lockdown counts.

## Introduction

In Fall 2020, severe acute respiratory syndrome coronavirus 2 (SARS-CoV-2) has Europe firmly in its grip again, and Switzerland, like its neighboring countries, is experiencing a strong second wave. Because the virus is now spreading again, it is important to provide better protection for people at elevated risk of developing severe courses of coronavirus disease 2019 (COVID-19). However, this can only be done if such risk groups are better defined and identified.

Over the last months, the evidence base has become increasingly strong that obesity, alongside with other relevant risk factors, is substantially and independently associated with adverse outcomes of COVID-19 ( Lockhart & O’Rahilly, 2020). A systematic review and pooled analysis of 75 studies recently confirmed that individuals with obesity were more at risk for COVID‐19 positive, for hospitalization, for ICU admission, and for mortality ( Popkin *et al.*, 2020). In the largest analyzed cohort study to date significantly increased risks of COVID-19-related death were seen for obesity grade 3 (body mass index (BMI) ≥40.0kg/m2) and grade 2 (BMI 35.0–39.9kg/m2), but not for obesity grade 1 (BMI 30.0–34.9kg/m2) and overweight (BMI 25.0–29.9kg/m2) ( Williamson *et al.*, 2020).

Beyond that, it has been shown that the association between obesity and COVID-19 is independent of diabetes and other diseases frequently associated with obesity and the metabolic syndrome, meaning that also so-called “healthy” obese people might be at elevated risk of severe COVID-19 ( Beretta, 2020; Tartof *et al.*, 2020). Switzerland seems to be no exception: For example, obesity was a significant part of the high burden of comorbidities in a sample of patients hospitalized with COVID-19 at the cantonal hospital Aarau (Switzerland) in Spring 2020 ( Gregoriano *et al.*, 2020). Thus, two pandemics are currently intertwining ( Lockhart & O’Rahilly, 2020), the communicable one of COVID-19 and the non-communicable one of obesity, both affecting significant parts of the population in Switzerland.

However, for Switzerland, due to the lack of synthesis studies, it is currently not clear how many people – in relative and absolute numbers – are actually affected by obesity at all, and thus have an increased risk of a severe COVID-19 course. There are a handful of individual representative population-based studies conducted between 2012 and 2017, but there are no overall estimates. Consequently, we aim to provide a first overall estimate of how many people in Switzerland are currently affected by grade 2 and 3 obesity. Such estimations may help to better estimate the relevance and size of this group at elevated risk, which could be incorporated into strategies to protect risk groups during the still unfolding COVID-19 pandemic.

## Methods

We accessed five representative national population-based studies which were conducted between 2012 and 2017 and which include data on height and weight of adult men and women in Switzerland ( Table 1). All five studies are based on representative samples and provide official sample weightings which allow to correct for non-participation. For all data sets we included only adults aged 18 years and older.

**Table 1.** Included representative studies.

Study	Acronym	Years	Age range	n
Swiss Health Survey ^1^	SHS12	2012	15–75	21597
Swiss Health Survey ^1^	SHS17	2017	15–75	22134
Swiss Household Panel ^2^	SHP	2013	14–85	5040
Swiss National Nutrition Survey ^3^	menuCH	2014/2015	18–75	2057
Statistics on Income and Living Conditions ^4^	SILC	2017	15–75	12980

^1^ https://www.bfs.admin.ch/bfs/de/home/statistiken/gesundheit/erhebungen/sgb.html ^2^ https://forscenter.ch/projects/swiss-household-panel/ ^3^ https://www.blv.admin.ch/blv/de/home/lebensmittel-und-ernaehrung/ernaehrung/menuch.html ^4^ https://www.bfs.admin.ch/bfs/en/home/statistics/economic-social-situation-population/surveys/silc.html

The **Swiss Health Survey (SHS)** is a representative survey of the health status, health behavior and use of health services of the Swiss population and has been conducted every five years since 1992. In the present study, the waves 2012 (SHS12, n=21,597 participants) and 2017 (SHS17, n=21,597 participants) were included. Both survey years were analyzed separately. A detailed description of the data collection, the recruitment procedure, the participation rate and the strategy of sample weighting was published elsewhere ( Bundesamt für Statistik, 2014; Storni *et al.*, n.d.). The SHS data were provided by the Federal Statistical Office (FSO). The **Swiss Household Panel (SHP)** is a representative longitudinal study of the social changes and living conditions of the Swiss population and has been conducted annually since 1999 with three samples (1999, 2004 and 2013). This study used cross-sectional data from the third sample from 2013 (n=5,040 participants). A detailed description of the data collection, the recruitment process, the participation rate and the strategy of sample weighting was published elsewhere ( Voorpostel *et al.*, 2020). The SHP data are available from the Swiss Centre of Expertise in the Social Sciences (FORS) website. **menuCH** is the first representative national nutrition survey of Switzerland conducted between 2014 and 2015 (n=2,057 participants). A detailed description of the data collection, weighting strategy, the recruitment process and the participation rate is published elsewhere ( Bochud *et al.*, 2017; Pasquier *et al.*, 2017). The data were provided by the Federal Food Safety and Veterinary Office (FSVO). The **Swiss Survey on Income and Living Conditions (SILC)** is a representative study of income and living conditions of Swiss households. Households are surveyed over several years and new households are added each year. This study uses cross-sectional data from 2017 (n=12,980 participants). A detailed description of the data collection, the recruitment procedure, the participation rate and the strategy of sample weighting was published elsewhere ( Schweiz. Bundesamt für Statistik BFS, 2014). The SILC data were provided by the Federal Statistical Office (FSO). Since all five data sets are publicly accessible for research purposes and are fully anonymized, no ethics permission was required for the present study.

With the exception of menuCH (where body height and body weight were also measured) the data sets contain exclusively self-reported height and weight information. Based on height and weight we calculated BMI (kg/m2). Very few people with a BMI <14.0 or >60.0 kg/m2 were excluded. Thereafter, BMI was categorized according to the WHO groups for underweight (BMI <18.5 kg/m2), normal-weight (18.5–24.9 kg/m2), pre-obesity (25.0–29.9 kg/m2), obesity grade 1 (30.0–34.9 kg/m2), obesity grade 2 (35.0–39.9 kg/m2), and obesity grade 3 (≥40.0 kg/m2) ( World Health Organization (WHO), 2020).

### Statistical analysis

The relative frequency or proportion of the BMI categories were calculated for each of the five studies. We used official sample weights provided by the data owners, but we did not adjust these estimated proportions for potential cofactors, mainly because we were interested in an overall estimation of the prevalence in Switzerland. To estimate the overall proportion we averaged the weighted proportions across all five data sets and calculated 95% confidence intervals via standard deviations. To estimate the absolute number of affected people in each of the BMI categories in all of the five studies individually we used official population numbers for adults in the corresponding survey years as denominator ( Schweiz. Bundesamt für Statistik BFS, 2020). To estimate the overall estimation of relative frequencies of the BMI groups we averaged the yearly population numbers 2012–2017. The statistical analyses were performed using R Version 3.6.0 ( R Core Team, 2018). Stata version 14 (Stata Corporation, College Station, TX, USA) was used for all analyses and graphs.

## Results

Our overall estimation shows that in Switzerland in 2012–2017, 31.72 % (95% CI 31.07-32.37) of the adult population were affected by overweight (BMI 25.0–29.9kg/m2) ( Table 2). Of the 11.20% who were obese (BMI ≥30.0kg/m2), 8.86% (95% CI 8.30-9.42) suffered from grade 1 obesity (BMI 30.0–34.9 kg/m2), 1.76% (95% CI 1.50-2.02) from grade 2 obesity (BMI 35.0–39.9 kg/m2), and 0.58% (95% CI 0.50-0.66) from grade severe 3 obesity (BMI>=40.0 kg/m2). Converted into estimated absolute population numbers, this corresponds to a total of approximately n=154,515 people who suffer from grade 2 or 3 obesity (n=116,216 and n=38,298, respectively). Overall, an estimated total of n=2,834,088 or 42.92% of the adult population in Switzerland are overweight or obese (BMI>=25.0 kg/m2).

**Table 2.** Overall estimation of the prevalence of World Health Organization (WHO) body mass index (BMI) categories across all 5 included representative data sets. To calculate the absolute numbers the average number of the adult residential population in Switzerland from the survey years 2012–2017 (n=6,603,188) was used as a denominator.

BMI WHO categories	Proportion in % (95% CI)	Estimated absolute number
underweight (<18.5)	2.82 (2.46- 3.18)	186210
normal (18.5 – 24.9)	54.28 (53.57-54.99)	3584211
pre-obesity (25.0 – 29.9)	31.72 (31.07-32.37)	2094531
obesity I (30.0 – 34.9)	8.86 (8.30- 9.42)	585042
obesity II (35.0 – 39.9)	1.76 (1.50- 2.02)	116216
obesity III (≥ 40.0)	0.58 (0.50- 0.66)	38298

Table 3 shows the convergence across the individually estimated relative frequencies from each of the five analyzed surveys. The proportion for grade 2 obesity ranged between 1.5% and 2.2%, and for grade 3 obesity between 0.5% and 0.7%. The highest relative frequencies for all three grades of obesity were calculated for the menuCH data.

**Table 3.** Estimation of the prevalence of World Health Organization (WHO) body mass index (BMI) categories for each of the 5 included representative data sets. The provided sample weights were used, but the estimations were not adjusted for cofactors.

BMI WHO categories	SHS 2012	SHS 2017	SHP 2013	menuCH 2014/2015	SILC 2017
underweight (<18.5)	3.3	2.9	2.9	2.3	2.7
normal (18.5 – 24.9)	54.7	55.2	54.3	53.9	53.3
pre-obesity (25.0 – 29.9)	31.9	31.2	31.3	31.4	32.8
obesity I (30.0 – 34.9)	8.1	8.5	9.4	9.4	8.9
obesity II (35.0 – 39.9)	1.6	1.7	1.5	2.2	1.8
obesity III (≥ 40.0)	0.5	0.5	0.6	0.7	0.6

SHS - Swiss Health Survey, SHP - Swiss Household Panel, menuCH - Swiss National Nutrition Survey, SILC - Statistics on Income and Living Conditions

**Figure 1.**
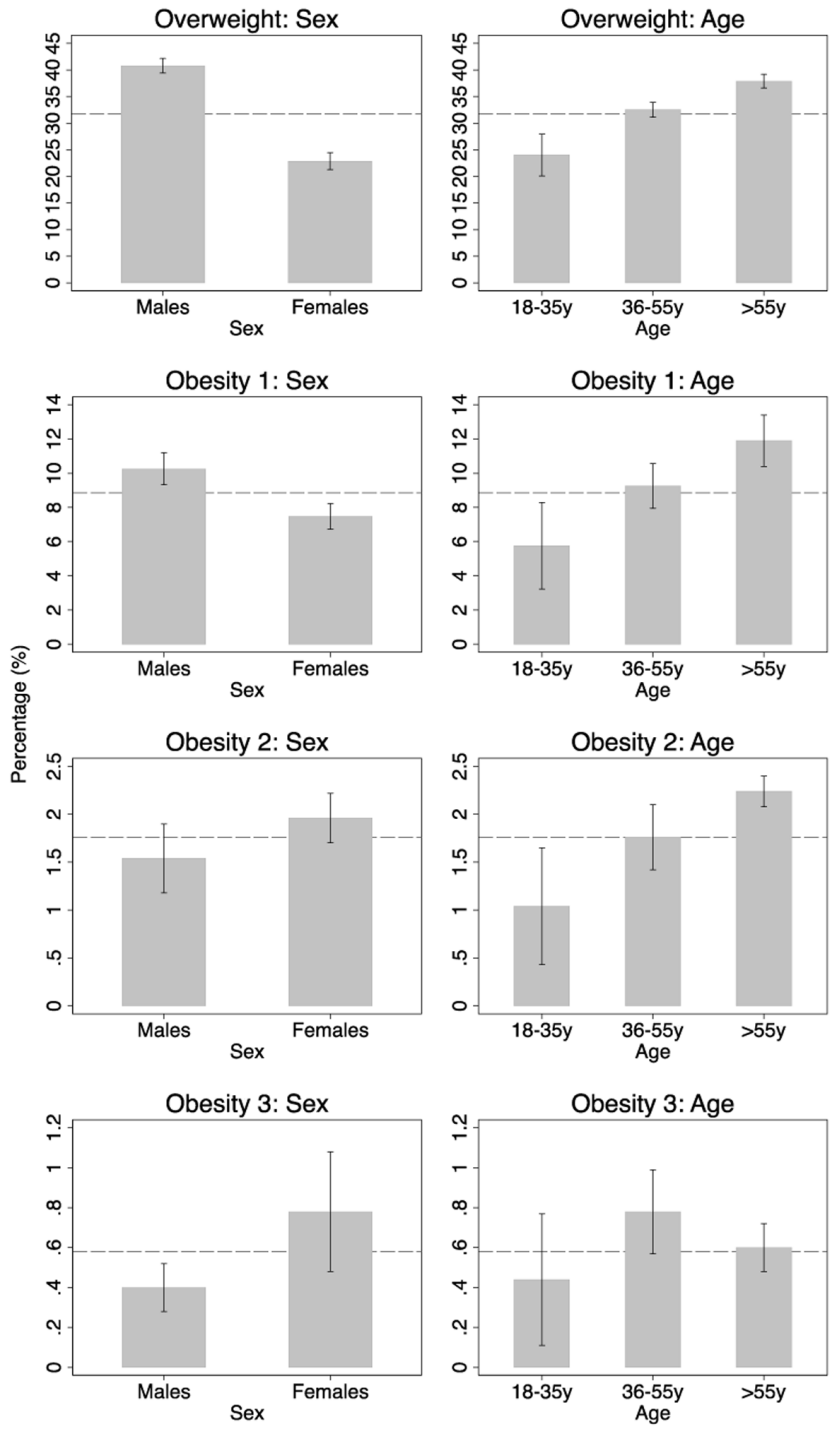
Overall estimation of the prevalence (incl. 95% CI) of World Health Organization (WHO) body mass index (BMI) categories across all 5 included representative data sets, stratified by sex (left column) and age groups (right column). The vertical dashed lines indicate the overall estimations from Table 1. The precise results can be found in Table 4.

The stratification by sex shows that men were more affected by overweight and grade 1 obesity ( Figure 1 and Table 4. However, for grade 2 and 3 obesity, there is a weak tendency towards women being slightly more affected. Although there is an increase in the proportion in all overweight and obesity (sub-)categories with increasing age, there are still considerable proportions of younger adults (aged 18–35 years) who suffer from obesity grade 2 (1.04%, 95%CI 0.43-1.65) or grade 3 (0.44%, 95%CI 0.11-0.77).

**Table 4.** Overall estimation of the prevalence of World Health Organization (WHO) body mass index (BMI) categories across all 5 included representative data sets, stratified by sex (A) and age groups (B).

A) Sex			
	Males	Females	
BMI WHO categories	Proportion in % (95% CI)	Proportion in % (95% CI)	
underweight (<18.5)	0.70 (0.50-0.90)	4.84 (4.08- 5.60)	
normal (18.5 – 24.9)	46.34 (44.06-48.62)	62.04 (60.09-63.99)	
pre-obesity (25.0 – 29.9)	40.76 (39.39-42.13)	22.86 (21.25-24.47)	
obesity I (30.0 – 34.9)	10.26 (9.32-11.20)	7.48 (6.73-8.23)	
obesity II (35.0 – 39.9)	1.54 (1.18-1.90)	1.96 (1.70-2.22)	
obesity III (≥ 40.0)	0.40 (0.28-0.52)	0.78 (0.48-1.08)	
B) Age groups			
	18–35y	36–55y	>55y
BMI WHO categories	Proportion in % (95% CI)	Proportion in % (95% CI)	Proportion in % (95% CI)
underweight (<18.5)	4.08 (2.25-5.91)	2.04 (1.45- 2.63)	2.08 (1.65- 2.51)
normal (18.5 – 24.9)	64.66 (58.93-70.39)	53.68 (51.38-55.98)	45.28 (43.10-47.46)
pre-obesity (25.0 – 29.9)	24.00 (20.06-27.94)	32.50 (31.11-33.89)	37.88 (36.60-39.16)
obesity I (30.0 – 34.9)	5.76 (3.23-8.29)	9.26 (7.96-10.56)	11.90 (10.39-13.41)
obesity II (35.0 – 39.9)	1.04 (0.43-1.65)	1.76 (1.42-2.10)	2.24 (2.08-2.40)
obesity III (≥ 40.0)	0.44 (0.11-0.77)	0.78 (0.57-0.99)	0.60 (0.48-0.72)

## Discussion

We show that the proportion of people in Switzerland who suffer from grade 2 or 3 obesity and thus an increased risk of severe COVID-19 course seems small in terms of percentages (2.34%), but when converted into absolute numbers it translates into a considerable number of human lives. At least 150,000 adults in Switzerland suffer from grade 2 or grade 3 obesity and are thus at elevated risk for adverse outcomes of COVID-19. This also includes many younger people in Switzerland.

It goes without saying that the causes of severe COVID-19 are only fragmentarily understood at this time ( Pairo-Castineira *et al.*, 2020) and that obesity frequently coincides with other risk factors for severe COVID-19 such as male sex, older age, diabetes, elevated blood pressure, and other comorbidities. However, recent studies suggest, that the association between obesity and severe COVID-19 is independent from these other cofactors, and experts debate diverse pathways and mechanisms which link both pandemics. It should be mentioned that an increased risk for disease severity among grade 3 obese people is already well documented for influenza, especially in past pandemics such as the Swine flu of 2009 ( Morgan *et al.*, 2010).

Among the possible explanations that link obesity with severe COVID-19 disease, the following pathways are currently mentioned in the literature: First, there are mechanical reasons which can worsen the prognosis in hospitals, as obesity is known to be associated with reduced lung volume/function, restricted airflow, and poor response to mechanical ventilation ( Caci *et al.*, 2020; Wadman, 2020). Second, obesity can be related to other complications, such as renal failure, cardiovascular dysfunction, hypertension, blood clotting, and vascular damage, which in turn can further influence negative outcomes of COVID-19 ( Caci *et al.*, 2020; Frühbeck *et al.*, 2020). Third, the biological and physiological pathways probably include obesity-driven chronic low-grade inflammation and metabolically dysregulated immune response to infection, which may drive organ injury in the development of severe COVID-19 and impair viral clearance ( Caci *et al.*, 2020; Chiappetta *et al.*, 2020; Frühbeck *et al.*, 2020; Huizinga *et al.*, 2020; Smith *et al.*, 2020; Wadman, 2020). However, other factors are currently proposed as well, such as links to fat embolism ( Cinti *et al.*, 2020) or growth hormone insufficiency ( Lubrano *et al.*, 2020). Finally, behavioral and social aspects could also play a role: People suffering from obesity may delay seeking medical care due to fear of being stigmatized which could increase their likelihood of severe COVID-19 ( Wadman, 2020). However, this would still have to be verified for Switzerland on the basis of hospitalization datas around the lockdown in spring 2020.

By October 2020, the Federal Office of Public Health declares that only people with grade 3 obesity (BMI>=40.0kg/m2, meaning weighting at least 126kg for an average tall man of 178cm or at least 111kg for an average tall women of 166cm) have an increased risk of severe COVID-19 ( Bundesamt für Gesundheit (BAG), 2020). However, this rather narrow definition of an obesity-related risk group probably needs to be extended based on recent evidence being published: In a large sample from Paris, all three grades of obesity doubled mortality in patients hospitalized with Covid‐19 ( Czernichow *et al.*, 2020), and in another large UK sample there was an upward linear trend in the likelihood of COVID-19 hospitalization with increasing BMI, that was evident already in overweight people (BMI 25.0–29.9kg/m2) ( Hamer *et al.*, 2020). The same pattern was documented in New York, where an increased risk of dying from COVID-19 was not only found in obese but also in overweight people with a BMI 25.0–29.9kg/m2 ( Nakeshbandi *et al.*, 2020). In our studies of large samples of conscripts for the Swiss Armed Forces, i.e. data with a high coverage for young and mostly healthy men, we show that, on the one hand, inflammatory blood parameters (CRP, leukocytes, neutrophils and basophils) ( Staub *et al.*, 2018) and, on the other hand, endurance performance (VO2max) ( Gassmann *et al.*, 2020) do not suddenly increase or decrease only with obesity (BMI ≥30.0kg/m2), but gradually increase with increasing BMI, even within the overweight BMI range (25.0–29.9kg/m2).

In sum, based on international studies, there is increasing evidence that grade 1 obesity and overweight are also associated with adverse COVID-19 outcomes. If this is confirmed, then significantly more people in Switzerland (approximately 585,042 with grade 1 obesity and 2,094,531 with overweight) would be exposed to this increased risk. However, as long as most studies virtually dichotomize BMI (obese vs. non-obese) and do not model it continuously and even non-linearly, it will remain unclear whether many more people with lighter forms of obesity and overweight are also at elevated risk. It would also be important in this context to go beyond BMI and also investigate other anthropometric proxies for body fat distribution, as it has recently been shown that patients with visceral adiposity or high intramuscular fat deposition have a particularly higher risk for critical COVID-19 illness and should be monitored more carefully when hospitalized ( Yang *et al.*, 2020).

Our study comes with limitations: First, the BMI is not an ideal measure of body composition because it cannot distinguish between muscle and fat in terms of weight. Nevertheless, at a population level, BMI is still strongly correlated with body fat ( Keys *et al.*, 1972; Kit *et al.*, 2014). Second, in four of the five studies included, information on height and weight was self-reported. In Switzerland, too, it has already been confirmed that men in particular report being taller than they are (especially with increasing age) and women, on the other hand, assess themselves less heavy ( Faeh *et al.*, 2009; Faeh *et al.*, 2008; Vinci *et al.*, 2019a). In both cases this would mean rather under- than overestimation of overweight and obesity levels in our analysis. In the case of the menuCH data, we observed that the overall proportions for the BMI categories according to WHO differ only marginally when comparing the measured with the self-reported BMI values. We did therefore not adjust our analysis for this potential bias. Third, population-based health surveys can be subjected to a healthy and higher education participation bias, meaning that participants in such surveys are generally slightly healthier and better educated than the general population ( Bopp *et al.*, 2014; Volken, 2013). The last two listed limitations, measured anthropometrics and participant bias might be among the reasons why in menuCH the proportions of overweight and obese participants was slightly higher than in the other four included surveys. Apart from the comparably small sample size and the relatively low participation rate (38%), one of menuCH's special features is that among the overweight (7.2%), and especially among the obese participants (14.1%), there are comparatively many people who have stated that they are on a weight-loss diet ( Vinci *et al.*, 2019b). This might be related to the fact that there was increased interest in a nutrition survey among these participants, which led to a slight increase in the participation rate, especially among obese people. However, the applied sample weights do not adjust for this participation bias.

## Conclusion

Because synthesis studies have been lacking until now, it was unclear how large the group of adults is in Switzerland, which has an increased risk of a severe COVID-19 course due to obesity. We have shown in this publication: This affects at least 150,000 adult people in Switzerland with grade 2 or 3 obesity. However, these numbers are 3.8 to 13.6 times higher if grade 1 obesity and overweight people are also included in this risk group, for which there are arguments arising in the latest literature. Relative and absolute numbers provide relevant but different perspectives. We think that especially in a pandemic, in which communication via numbers is so dominant, it is important not to lose the relation to the absolute numbers, especially when it concerns human lives.

The health emergency caused by the COVID-19 pandemic currently diverts attention from the prevention and care of non-communicable chronic diseases, such as obesity, to communicable diseases ( Dicker *et al.*, 2020). It is important to focus increasingly on the interlinks between the two pandemics, because obviously in this case a communicable and a non-communicable pandemic are linked. Professional societies such as European Association for the Study of Obesity (EASO) are currently developing position statements and guidelines on the manifold challenges for obese people during this COVID-19 pandemic ( Frühbeck *et al.*, 2020).

We think that two fields of action are particularly important: A) Current NCD prevention programs aimed at preventing weight gain in the general population should be continued or even intensified, despite the current focus on infectious diseases. B) The numerous people who also suffer from obesity in Switzerland should be given more attention and support. This applies not only to obese people who are infected with the virus, but to this numerous group in general, as stigmatization, lockdowns, stress, anxiety, or isolation have many consequences for health behaviors and well-being on various levels, especially in such vulnerable groups with preconditions ( Frühbeck *et al.*, 2020). A particular challenge for such public health programs at various levels is certainly that overweight and obese people are not a homogenous group, as we have shown in our previous work ( Vinci *et al.*, 2019b), and specific subgroups might need specifically tailored approaches. Regionally and socially differentiated public health strategies are very important in identifying these risk groups, especially if one has to react quickly and with limited resources. However, the effort might be worth it: If the current scientific state of knowledge becomes more and more solid and obesity really does play that major role in severe COVID-19 courses, then every kilo of body weight that is not gained in lockdowns or that is lost counts ( Beretta, 2020). It might even be doubly worth the effort, as obesity is known to be a risk factor for other factors such as cardiovascular diseases or diabetes, which are themselves (independently?) associated with increased risk of severe COVID-19 courses.

## Data availability

### Source data

The five data sets on which this paper is based are owned and administered by the Federal Food Safety and Veterinary Office (FSVO) and the Federal Office of Public Health (FOPH). Based on signed data contracts the authors of this paper are not authorized to share the individual data. However, all studies used can be accessed by other researchers via standard procedures requested by the FSVO (webpage menuCH), the FOPH (webpage SILC, webpage SHS), and FORS (webpage SHP), thus all results of this paper can easily be replicated.
