# Supplementary material for: Clash of the pandemics – At least 150’000 adults in Switzerland suffer from obesity grades 2 or 3 and are thus at elevated risk for severe COVID-19
Source: F1000Res. 2020 Dec 7;9:1413. [Version 1] doi: 10.12688/f1000research.27819.1 (PMC7970431; doi:10.12688/f1000research.27819.1)
Supplement: Peer review history

# Peer review: Reviewer response for version 1

**DOI:** 10.5256/f1000research.30764.r79811

**Published:** 2021-03-17

**Authors:** Jon D Samuels

**Affiliations:** 1 Department of Anesthesiology, Weill Cornell College of Medicine, New York-Presbyterian Hospital, New York, New York, USA

In this timely and well written publication, Staub and colleagues connect the obesity epidemic with the emerging SARS-CoV-2 epidemic. The paper is peppered with targeted and timely references. One would hope that parallel investigations would emerge from other countries.

 What remains to be investigated in future publications is an in-depth analysis of the obese population in both the first and third world, to determine the statistical variation in both the co-morbidity load and the SARS-CoV-2 disease representation.

Is the work clearly and accurately presented and does it cite the current literature?

Yes

If applicable, is the statistical analysis and its interpretation appropriate?

Yes

Are all the source data underlying the results available to ensure full reproducibility?

Yes

Is the study design appropriate and is the work technically sound?

Yes

Are the conclusions drawn adequately supported by the results?

Yes

Are sufficient details of methods and analysis provided to allow replication by others?

Yes

Reviewer Expertise:

Morbid obesity, obstructive sleep apnea, and bariatric surgery. The adult difficult airway. Airway management equipment and supplies.

I confirm that I have read this submission and believe that I have an appropriate level of expertise to confirm that it is of an acceptable scientific standard.

# Peer review: Reviewer response for version 1

**DOI:** 10.5256/f1000research.30764.r79863

**Published:** 2021-02-22

**Authors:** Carlos Alberto Nogueira-de-Almeida

**Affiliations:** 1 Departamento de Medicina, Universidade Federal de São Carlos, São Carlos, Brazil

The study seeks to detect, in the Swiss population, the number of people who would be at risk for severe COVID-19 based on their body mass index. The idea is based on information that obese grades 2 and 3 are at high risk, and thus the goal is to raise how many people in the country would belong to this risk group. This rational makes perfect sense.

 It is a simple study, which is based on data already published and makes an estimate only of the prevalence of obesity. The link with COVID-19 is just that, but taking into account the severity of the present pandemic, all possible information are important and relevant. The rational of the study is very well written. Considering the merits of collecting data that may be useful for strategies to cope with the disease in Switzerland and the simple but careful methodology, indexing is justified.

A simple suggestion:

 In the abstract, the first sentence of the conclusion refers to the fact that the risk group does not include many young people. In fact, this is relevant information, but only a secondary conclusion and should not appear prominently in the first sentence.

Is the work clearly and accurately presented and does it cite the current literature?

Yes

If applicable, is the statistical analysis and its interpretation appropriate?

Yes

Are all the source data underlying the results available to ensure full reproducibility?

Yes

Is the study design appropriate and is the work technically sound?

Yes

Are the conclusions drawn adequately supported by the results?

Partly

Are sufficient details of methods and analysis provided to allow replication by others?

Yes

Reviewer Expertise:

Nutrition and Obesity

I confirm that I have read this submission and believe that I have an appropriate level of expertise to confirm that it is of an acceptable scientific standard.
